# Approaches to visualize, quantify, and manipulate phosphoinositides in cells

**DOI:** 10.1007/s00418-026-02479-5

**Published:** 2026-05-05

**Authors:** Rabia Gönül, Agnieszka Chytła, Ana Miladinović, Ludovica Antiga, Pavel Hozák, Martin Sztacho

**Affiliations:** 1 Laboratory of Cancer Cell Architecture, Institute of Biochemistry and Experimental Oncology, First Faculty of Medicine, Charles University, Prague, Czech Republic; 2 Department of Biology of the Cell Nucleus, Institute of Molecular Genetics of the Czech Academy of Sciences, Prague, Czech Republic

**Keywords:** Phosphoinositides, Lipid signaling, Subcellular compartmentalization, Cellular architecture, Live-cell imaging, Lipid quantification

## Abstract

Phosphoinositides are low-abundance regulatory lipids that control a broad range of cellular processes, from membrane trafficking and cytoskeletal remodeling to transcriptional regulation and RNA processing. These lipids are distributed across distinct subcellular compartments, where they carry out compartment-specific regulatory functions. Dysregulation of phosphoinositide metabolism is associated with cancer, neurodegenerative diseases, and immune dysfunction. However, their roles remain difficult to investigate owing to technical limitations in lipid detection and manipulation. This review outlines current strategies for modulating, visualizing, and quantifying phosphoinositide pools, including genetic manipulation techniques such as RNA interference, clustered regularly interspaced short palindromic repeats (CRISPR)-based approaches, and optogenetics. It also evaluates visualization tools such as fluorescent biosensors and live-cell imaging techniques, including superresolution microscopy. In parallel, quantitative methods such as thin-layer chromatography and mass spectrometry for profiling phosphoinositide species, including isomer- and acyl-specific variants, are discussed. By comparing the strengths and limitations of these approaches and highlighting how they can be combined, this review provides a practical framework for dissecting phosphoinositide function in defined subcellular contexts.

## Introduction

Phosphoinositides are members of the glycerophospholipid family present in both eukaryotes and prokaryotes (Beresford et al. 2010; Botero et al. 2019; de Azevedo-Martins et al. 2021). They are derived from a common phosphatidylinositol (PI) scaffold and differ in the number and position of phosphate groups on the inositol headgroup, forming seven different phosphoinositide species (Casalin et al. 2024). These lipids are distributed across distinct cellular compartments and regulate key cellular processes such as signal transduction, cytoskeletal organization, and apoptosis (Balaban et al. 2023; Balla 2013; Castano et al. 2019; Saarikangas et al. 2010; Vidalle et al. 2023). Beyond their well-established cytoplasmic and membrane-associated roles, phosphoinositides are also detected in the nucleus, where they form localized pools within distinct nuclear compartments associated with RNA polymerase I/II-dependent transcription and RNA processing (Balaban et al. 2023, 2021; Chytla et al. 2025; Hoboth et al. 2024; Sztacho et al. 2021).

Phosphoinositide levels are tightly regulated by lipid kinases, phosphatases, and phospholipases, and dysregulation of these enzymes contributes to a wide range of diseases, including neurodegenerative disorders, immune dysfunction, metabolic diseases, and cancer (Balla 2013; Raghu et al. 2019). Mutations in phosphatase and tensin homolog (PTEN) and in the catalytic subunit α of phosphatidylinositol 3-kinase (PIK3CA) are among the most frequently observed alterations in many cancer types. Such mutations disrupt the balanced regulation of phosphoinositide pools, often resulting in elevated levels of PI(3,4,5)P3 and consequent hyperactivation of the phosphoinositide 3-kinase/protein kinase B (PI3K/Akt) signaling pathway. Sustained activation of this pathway promotes uncontrolled cell proliferation and survival, thereby driving tumorigenesis (Madsen et al. 2018; Mendes-Pereira et al. 2009; Porta et al. 2014; Zou et al. 2020). Understanding how phosphoinositides and their regulatory enzymes govern cellular functions is therefore essential for elucidating disease mechanisms.

Despite numerous studies investigating phosphoinositides across diverse cellular processes, their dynamics within and between cellular compartments remains difficult to resolve. This challenge arises from their low cellular abundance, rapid turnover, and highly localized distribution, such that even subtle changes can produce pronounced biological effects while remaining below the detection limits of individual experimental approaches. Moreover, any single method provides only a partial view of phosphoinositide behavior. Consequently, resolving small and rapidly changing phosphoinositide pools requires experimental strategies that combine complementary techniques rather than relying on a single approach. This review summarizes current and emerging approaches for manipulating, quantifying, and visualizing phosphoinositides and discusses how their integrated application improves experimental control and data interpretation.

## Tools for phosphoinositide level manipulation

Understanding how individual phosphoinositide (PIP) species contribute to cellular functions requires approaches that can distinguish lipid-specific effects from broader changes in the membrane lipid pool. This is commonly achieved by manipulating phosphoinositide-metabolizing enzymes, as altering their activity or expression enables controlled perturbation of defined PIP species.

### Small-molecule modulators

**Fig. 1:**
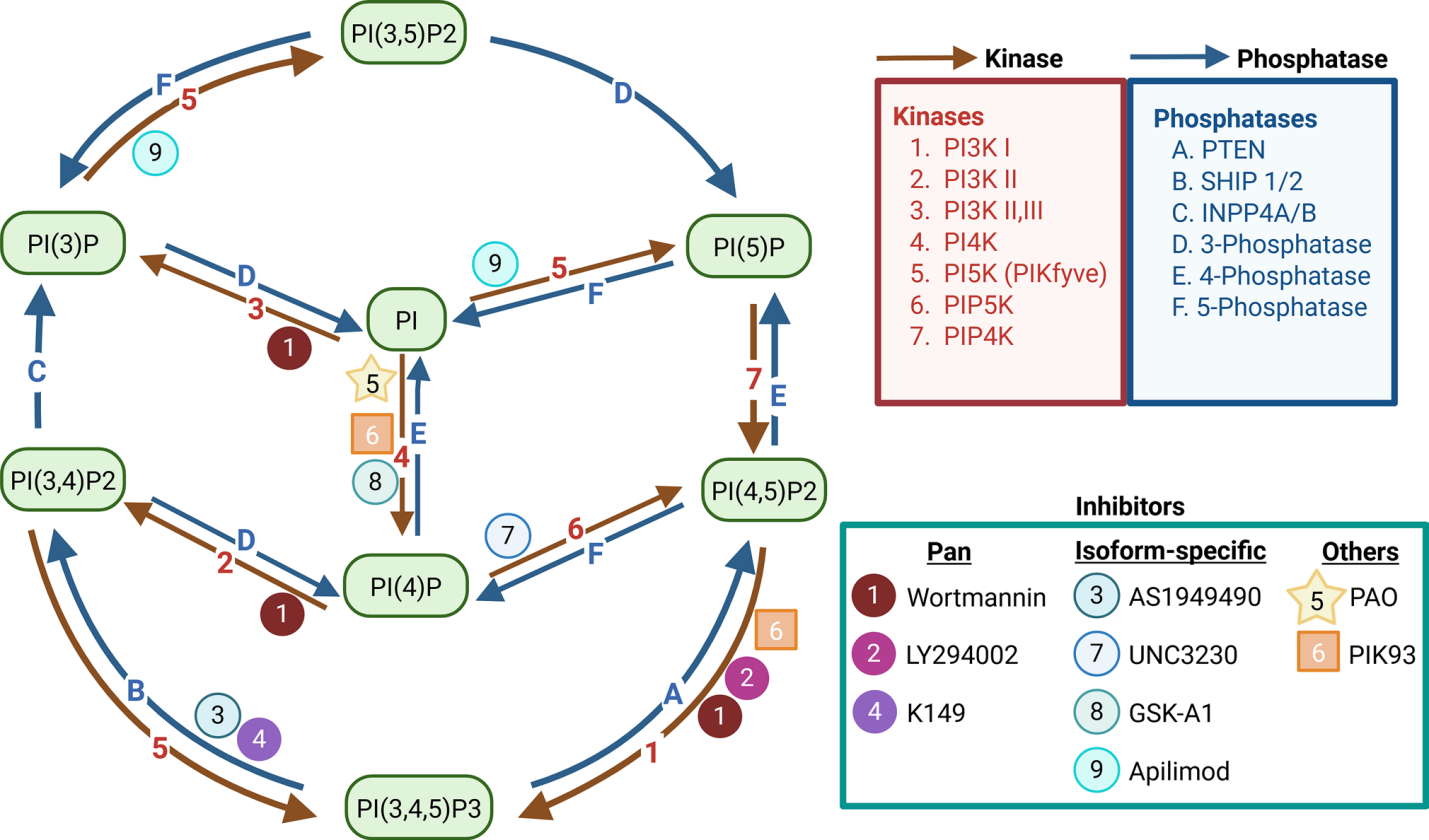
Overview of phosphoinositide metabolic pathways and the steps targeted by selected inhibitors. Kinase- and phosphatase-driven enzymatic steps linking distinct phosphoinositide species are indicated in red and blue, respectively. Enzymatic steps are labeled by numbers (kinases) and letters (phosphatases) referring to the legend. Small-molecule inhibitors (1–8) are mapped to the reactions they block. Created in https://BioRender.com

Among the strategies used to modulate enzymes in the phosphoinositide pathway, small-molecule inhibitors represent a well-established approach (Fig. 1). Their rapid inhibitory kinetics, often observable within minutes, offer the temporal resolution necessary to monitor dynamic changes in phosphoinositide pools (Arcaro and Wymann 1993; Ng et al. 2001; Zeng et al. 2016). In practice, two major classes of inhibitors are commonly employed: broad-spectrum pan-inhibitors that act on multiple isoforms, and isoform-selective inhibitors designed to preferentially target individual catalytic variants (Arcaro and Wymann 1993; Ng et al. 2001). Representative inhibitors, their main targets and mechanism of action are summarized in Table 1.

**Table 1:** Selected small-molecule modulators targeting phosphoinositide metabolism

Name	Target	Selectivity (pan-, isoform-specific, etc.)	Mechanism (reversible versus irreversible)	Typical working concentration (cells)	Ref.
Wortmannin	PI3K (catalytic subunit of PI3K)	Pan-inhibitor	Irreversible	~ 10–200 nM	(Balla et al. 2005; Zhang et al. 2003)
	PI4KIIIα			≥ ~ 10 µM	
LY294002	PI3K (ATP-binding site)	Pan-inhibitor	Reversible	1–50 µM	(Gharbi et al. 2007; Walker et al. 2000)
AS1949490	SHIP2	Isoform-specific	Reversible	0.3–10 µM	(El Sayed et al. 2024; Suwa et al. 2009)
K149	SHIP1/SHIP2	Pan-inhibitor	Likely reversible	0.5–10 µM	(Pedicone et al. 2020; Sztacho et al. 2024)
PAO	PI4K	Nonselective; reacts with many cysteine-containing enzymes	Irreversible under cell-culture conditions, however can be chemically reversed by thiol-reducing agents	1–10 µM	(Hammond et al. 2012; Harraz et al. 2018; Sun et al. 2009)
PIK93	PI4KIIIβ PI3Kα/γ/δ/β	Multitarget; strong inhibition of PI4KIIIβ, cross-inhibition class I PI3K	Reversible	0.05–1 µM	(Knight et al. 2006; Lin et al. 2023Vishakantegowda et al., 2024)
UNC3230	PIP5K1C (PIPKIγ)	Isoform-specific	Reversible	0.5–1 µM	(Seo et al. 2024; Wright et al. 2014)
GSK-A1	PI4KA (PI4KIIIα)	Isoform-specific	Reversible	10–100 nM	(Bojjireddy et al. 2014; Sengupta et al. 2019)
Apilimod	PIKfyve PI5P	Isoform-specific	Reversible	10–100 nM	(Cai et al. 2013; Sbrissa et al. 2018)

^*^Listed compounds are representative examples. Their activity depends on the experimental setup and cell type

Beyond blocking enzyme activity, isoform-selective inhibitors reveal pathway robustness and define the contribution of individual enzyme isoforms to phosphoinositide production (Balla et al. 2005; Costa et al. 2015). Selective inhibition of PI3Kα by BYL719 (alpelisib) in breast cancer cells illustrates pathway robustness, as prolonged treatment induces compensatory PI3Kβ activation and restores PI(3,4,5)P3 levels after approximately 24 h (Costa et al. 2015). Such adaptive responses highlight the need to consider inhibitor behavior in a cellular context. Given the differences in enzymatic target and specificity, determining appropriate dosing and timing must be carefully optimized for each cell type, particularly since some inhibitors lose selectivity at higher concentrations (Gharbi et al. 2007; Ng et al. 2001). For example, wortmannin acts as a potent PI3K inhibitor at nanomolar concentrations, yet at micromolar concentrations it loses specificity and inhibits phosphatidylinositol 4-kinase type IIIα (Balla et al. 2005; Gharbi et al. 2007; Walker et al. 2000).

In addition to concentration-dependent effects, the complexity of phosphoinositide metabolism sometimes necessitates simultaneous perturbation of multiple pathway nodes. Another way to induce a pronounced shift in a specific lipid pool is to target multiple steps within a phosphoinositide pathway. For instance, coordinated inhibition of PI4K by PAO and PIK93, and PIP5K by UNC3230, resulted in a markedly stronger reduction of PI(4,5)P2 levels than inhibition of any single enzyme alone (Harraz et al. 2018). Importantly, small-molecule inhibitors are not limited to kinases, as lipid phosphatases can also be targeted. A clear illustration of phosphatase targeting was provided by the inhibition of SH2 domain-containing inositol 5-phosphatase 2 (SHIP2) with K149, which effectively increased nuclear PI(4,5)P2 levels (Sztacho et al. 2024). Since chemical inhibitors can produce off-target effects that activate unintended pathways, genetic manipulation of the target enzyme offers a longer-term counterpart to these acute changes in lipid levels and helps verify whether observed changes arise from the intended mechanism.

### Genetic approaches

**Fig. 2:**
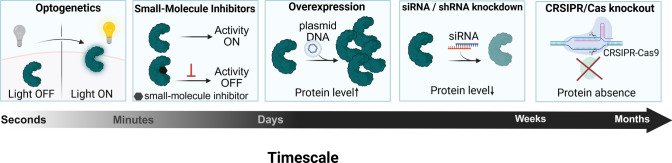
Short- and long-term approaches to phosphoinositide manipulation. Experimental strategies to manipulate phosphoinositide levels differ markedly in the onset and persistence of their effects. Optogenetic and chemically inducible systems provide rapid and reversible control on the scale of seconds to minutes, enabling interrogation of acute and spatially restricted lipid signaling. Small-molecule inhibitors act within minutes and are well suited for transient perturbation of enzymatic activity. In contrast, protein overexpression, RNA interference, and CRISPR-Cas-based genome editing induce slower but sustained changes in phosphoinositide metabolism, with functional consequences emerging over days to weeks. Together, these approaches span complementary temporal windows and should be selected according to the dynamics of the phosphoinositide process under investigation. Created in https://BioRender.com

Unlike small-molecule modulators, genetic approaches allow stable and highly specific manipulation of phosphoinositide enzymes, making it possible to follow changes in their expression or function over time and to uncover isoform-specific functions and slower cellular adaptation mechanisms (Fig. 2). Several genetic approaches are available to achieve this, including RNA interference (RNAi), clustered regularly interspaced short palindromic repeats (CRISPR), overexpression, and inducible or optogenetic systems.

#### Gene silencing

RNAi-mediated knockdown mirrors the effects of small-molecule inhibitors by decreasing enzyme activity, yet it also reduces protein expression. As knockdown requires time for messenger RNA (mRNA) and protein depletion, the resulting changes become apparent over a period of hours to days (Miladinovic et al. 2025; Moore et al. 2010; Sun et al. 2011). Several RNAi-based strategies can be used to achieve this, each differing in onset and duration of gene silencing. This approach enables more controlled modulation of specific phosphoinositide pools and systematic assessment of downstream structural or signaling responses. Two RNAi-mediated knockdown methods are widely used: small interfering RNA (siRNA) and short hairpin RNA (shRNA). Although both approaches can produce similar outcomes, they differ in how quickly silencing occurs and in how long the effect is maintained (Subramanya et al. 2010).

In the case of siRNA, knockdowns are rapid and transient, typically lasting up to 5 days (Subramanya et al. 2010). A representative example of the usage of this method in phosphoinositide research is depletion of PIP5K1A or SHIP2, to shift nuclear PI(4,5)P2 levels and investigate the role of PI(4,5)P2 in nuclear organization (Miladinovic et al. 2025; Sztacho et al. 2024). This short-lived yet fast-acting nature makes siRNA particularly useful for experiments requiring acute perturbation without long-term compensatory adaptation. In contrast, shRNA produces a more stable and long-term knockdown, maintaining gene silencing for weeks to months, and can even be used to generate stable knockdown cell lines (Subramanya et al. 2010). The ability of shRNA to maintain prolonged gene suppression makes it particularly valuable for dissecting isoform-specific enzymatic functions and slower cellular adaptation mechanisms that unfold over extended timescales. This has been illustrated in the sustained knockdown of PIP5Kγ in Jurkat T cells (Sun et al. 2011). Thus, while siRNA enables rapid and transient silencing, shRNA supports long-term studies where persistent pathway modulation is necessary. Although their mechanisms and effect durations differ, gene-silencing approaches reduce enzyme levels gradually rather than eliminating them entirely, allowing lipid pool changes and associated cellular responses to be examined under conditions that maintain pathway function.

#### Gene editing

Gene editing provides a complementary approach to RNAi. While shRNA achieves stable knockdown by reducing mRNA levels, yielding partial and tunable suppression, CRISPR-based approaches can eliminate gene function entirely through targeted genomic disruption, enabling analysis of long-term phenotypes and compensatory network responses (Barrangou et al. 2015). This distinction is illustrated by studies in which siRNA was used for acute analysis, while CRISPR/CRISPR-associated protein 9 (Cas9) generated stable knockout lines were used to uncover sustained regulatory effects (Malek et al. 2017). While CRISPR–Cas9 enables precise and efficient gene editing, traditional transgenic approaches still offer advantages in situations where CRISPR cannot be applied or where large-scale, organism-level modifications are necessary (Lampreht Tratar et al. 2018; Liu et al. 2017). By altering enzymes that regulate upstream metabolic precursors, these methods can shift phosphoinositide metabolism globally. This provides a level of pathway modulation that single-gene CRISPR edits cannot easily achieve and avoids the complexities of targeting individual kinases or phosphatases (Caires et al. 2021).

#### Gene and protein manipulation

Whereas gene silencing and editing provide loss-of-function perspectives, protein overexpression offers a gain-of-function approach for examining phosphoinositide regulation (Keselman et al. 2007; Sun et al. 2011). In addition to the manipulation of phosphoinositide enzymes, overexpression can also be applied to phosphoinositide-binding proteins that are not enzymatic components of the pathway (Kwon et al. 2009; Li et al. 2016; Sagawa et al. 2003). For instance, increasing levels of gelsolin, a high-affinity PI(4,5)P2-binding regulator of actin dynamics, impaired PI(4,5)P2 hydrolysis and altered protein kinase C (PKC) signaling, leading to decreased proliferation, tumorigenicity, and invasive behavior in carcinoma models (Li et al. 2016; Sagawa et al. 2003). However, overexpression results should be interpreted carefully, as it produces a fixed increase in expression and may introduce secondary effects that influence interpretation (Bolognesi & Lehner 2018). For this reason, overexpression is often combined with complementary approaches, such as small-molecule modulators or siRNA-mediated depletion, to verify that the observed phenotypes reflect perturbations within the phosphoinositide pathway (Keselman et al. 2007; Kwon et al. 2009).

In addition to combining methods, another way to overcome the limitations of fixed expression levels is to use inducible systems, which enable regulated and reversible expression. By responding to external cues such as small molecules or light, these systems allow precise control over both the timing of induction and the duration of the response (Kallunki et al. 2019; Siddiqui et al. 2022). Induction can be triggered chemically, via tetracycline, tamoxifen, or rapamycin, or nonchemically through recombinase action or light-based activation (Kallunki et al. 2019). Among inducible approaches, tetracycline-based systems are widely used because they enable adjustable and reversible control of gene expression. In Tet-off systems, a TetR-derived transactivator binds the promoter and drives transcription until tetracycline or doxycycline is added, whereas Tet-on systems use a reverse TetR mutant that activates transcription only in the presence of the drug (Forster et al. 1999; Rang & Will 2000). This design makes them suitable for experiments that require timed expression of phosphoinositide-related enzymes or binding proteins. In this context, tetracycline-inducible systems allow controlled modulation of phosphoinositide levels by regulating enzyme expression, helping to isolate effects mediated by specific lipid–protein interactions (Chi et al. 2023). A common limitation of these systems is basal leakiness, in which the gene is expressed at low levels even when it is intended to be turned off. However, newly optimized designs help suppress this unwanted background activity, thereby improving the precision of inducible control (De Carluccio et al. 2024).

Although small-molecule inducers provide valuable temporal control, optical systems offer an added level of precision by enabling activation at specific sites within the cell. Light-based inducible systems use genetically encoded photosensitive domains, such as cryptochrome 2 (CRY2) with the membrane-anchored N-terminal domain of CIB (CIBN) or light–oxygen–voltage (LOV)-based modules, that change their conformation or binding behavior upon illumination (Emiliani et al. 2022; Kallunki et al. 2019; Zhu et al. 2025). Through this light-driven control, these systems are well-suited for studying phosphoinositide dynamics because light-triggered recruitment or activation of kinases and phosphatases enables real-time, reversible control of specific lipid pools (Idevall-Hagren et al. 2012; Tan et al. 2022; Ueda et al. 2022). Even though these optogenetic tools are highly precise, careful analysis is required owing to limitations such as spectral overlap with fluorescent reporters, mild basal activity, and potential phototoxicity during illumination (Idevall-Hagren et al. 2012; Tan et al. 2022; Ueda et al. 2022). Taken together, small-molecule inhibitors are an efficient tool for rapidly altering phosphoinositide metabolism and remain the most practical initial approach. Genetic methods, in contrast, reveal the consequences of sustained changes in enzyme levels, including pathway adjustments that emerge over longer timescales. Inducible and optogenetic systems bridge these approaches by allowing localized, tunable, and reversible perturbations, making them particularly well-suited for probing compartment-specific and dynamic phosphoinositide regulation. Together, these manipulation strategies clarify how individual components shape phosphoinositide behavior. To address the limitations, these methods are often paired with quantitative analytical techniques. Mass spectrometry (MS) enables detailed profiling of lipid composition, while high-performance liquid chromatography (HPLC) provides measurements of changes in lipid abundance. Fluorescence-based imaging approaches further permit real-time visualization of phosphoinositide dynamics in living cells.

## Multimodal approaches to phosphoinositide quantification

Manipulating phosphoinositide pathways shows how they can be altered, but understanding what those changes mean requires sensitive quantitative methods. Having outlined the tools used to perturb the pathway, we now turn to the techniques that measure the resulting effects. Biochemical assays provide direct quantification of phosphoinositide levels by determining the abundance of individual lipid species. Chromatography-based separation and MS further refine this analysis by resolving and quantifying distinct phosphoinositides with varying degrees of selectivity, meaning that the choice of method directly shapes the obtained lipid profiles.

### Thin-layer chromatography

Although more advanced analytical techniques have become available, thin-layer chromatography (TLC) continues to play an important role in phosphoinositide research owing to its simplicity and accessibility. In this method, lipids are applied to silica gel plates and resolved by organic solvent systems according to differences in polarity and charge. For phosphoinositides, these properties correlate with the number of phosphates on the inositol headgroup, allowing distinct species to be separated (Furutani et al. 2006; Santiago and Strobel 2013). Classical phosphoinositide TLC workflows often rely on radiolabeling, using myo-[^3^H]inositol or [^32^P]orthophosphate, to enable sensitive detection of individual lipid species (Serunian et al. 1991). A practical nonradioactive alternative is liquid chromatography blotting (LC blotting), in which lipids separated by TLC are transferred onto polyvinylidene fluoride (PVDF) membranes and detected using tagged lipid-binding domains (e.g., GST-tagged domains), allowing visualization of multiple phosphoinositide species from a single plate (Furutani et al. 2006).

Through these applications, TLC provides a versatile and cost-effective approach for semiquantitative analysis of phosphoinositide abundance, enzymatic specificity, and pathway responses. Moreover, TLC can be combined with more quantitative and analytical techniques to expand the information obtained from a single separation. Following TLC separation, gas chromatography can be used to analyze both inositol headgroups and fatty acyl chains (Konig et al. 2008). Similarly, TLC can be coupled with mass spectrometry (MS), either through direct in situ ionization from the plate or indirectly by scraping and extracting separated spots prior to MS analysis (DeLong et al. 2001; Engel and Schiller 2021; Teuber et al. 2010). These combinations increase the information gained from a single separation by coupling TLC-based separation with MS-based identification of individual lipid species (Engel and Schiller 2021; Wenk et al. 2003). These hybrid approaches link TLC-based lipid class separation with MS-based molecular identification, substantially increasing analytical depth (DeLong et al. 2001; Engel and Schiller 2021; Serunian et al. 1991; Wenk et al. 2003).

### Mass spectrometry

MS is a broadly applied analytical technique used to identify and quantify biomolecules on the basis of the mass-to-charge ratios of ionized species. In lipid research, MS has become a central tool for resolving molecular composition and abundance across diverse lipid classes. When applied to phosphoinositide analysis, MS enables identification of individual species, estimation of their relative abundance, and characterization of both phosphorylation state and acyl-chain composition. However, many phosphoinositides occur at low abundance and share overlapping precursor masses, limiting the resolving power of single-stage MS for closely related species (Kim et al. 2010; Milne et al. 2005). Consequently, reliable detection depends on preceding extraction methods that preserve these highly polar lipids, as well as optimized ionization conditions that generate strong and stable signals (Milne et al. 2005).

Electrospray ionization tandem mass spectrometry (ESI-MS/MS) overcomes some limitations of single-stage MS by fragmenting selected precursor ions to generate headgroup- and acyl-chain-specific fragments. These product ions provide information on phosphate content and fatty-acyl composition, allowing the resolution of phosphoinositide species that cannot be distinguished by precursor mass alone (Milne et al. 2005). Despite this improvement, ESI-MS/MS still cannot fully resolve positional isomers or species with highly similar acyl-chain patterns.

More recent methodological advances have addressed this limitation by combining phosphate methylation with chiral chromatographic separation and ESI–MS using data-independent acquisition strategies such as sequential window acquisition of all theoretical fragment ion spectra (SWATH). This approach enabled clear discrimination among PI3P, PI4P, and PI5P, as well as between PI(3,4)P2, PI(3,5)P2, and PI(4,5)P2, while simultaneously providing detailed acyl-chain profiling (Li and Lammerhofer 2021; Llorente et al. 2023). As chromatographic and MS acquisition strategies have continued to evolve, phosphoinositide analysis has shifted from bulk quantification toward increasingly detailed molecular and spatial characterization. Coupling MS with mass spectrometry imaging (MSI) places these measurements into a spatial context, enabling direct mapping of phosphoinositide species within intact tissues (Barneda et al. 2025). In parallel, workflows such as phosphoinositide regioisomer measurement by chiral chromatography and mass spectrometry (PRMC-MS) allow simultaneous analysis of all seven phosphoinositide classes across their regioisomeric and acyl-chain variants, facilitating detection of changes in abundance, isomer distribution, and acyl-chain composition in both intracellular and extracellular pools. Finally, MS-based interactomics complements these approaches by identifying proteins that associate with specific phosphoinositides, linking lipid composition to functional signaling complexes (Mazloumi Gavgani et al. 2021).

Taken together, TLC efficiently resolves major phosphoinositide classes and is compatible with radiolabeling, lipid-binding probes, and a range of downstream analytical workflows. Although its limited sensitivity and inability to distinguish positional isomers restrict its quantitative power, TLC remains a widely used and practical approach for initial phosphoinositide separation, semiquantitative assessment of lipid pool changes, and validation of lipid identities. Moreover, TLC may be applied in combination with MS-based analyses. In contrast, MS enables detailed characterization of phosphoinositides in terms of abundance, phosphorylation state, and acyl-chain composition, with recent methodological advances allowing discrimination of closely related species, including regioisomers. Improvements in extraction, derivatization, and chromatographic separation have increased detection sensitivity for these low-abundance lipids, while integration of MS with imaging and protein-interaction analyses extends measurements to spatial distribution and lipid-associated signaling complexes. Together, TLC and MS provide complementary semiquantitative and quantitative readouts that support experiments aimed at manipulating phosphoinositide levels and interrogating pathway dynamics.

## Multimodal approaches to phosphoinositide visualization

A comprehensive understanding of phosphoinositides requires more than quantitative data alone. While quantitative methods provide precise measurements and can detect subtle changes in phosphoinositide levels, they offer limited information about rapid signaling events or intracellular localization. Fluorescence-based imaging fills this gap by enabling real-time visualization of phosphoinositides and their spatial and temporal behavior in living cells.

### Molecular biosensors

Selecting an appropriate biosensor is a critical first step in phosphoinositide visualization. The most widely used tools are recombinant biosensors, which rely on phosphoinositide-binding protein domains fused to fluorescent tags (e.g. GFP, YFP, mCherry), nonfluorescent tags (e.g. Snap-tag, Halo-tag, GST), or luciferase (Crivat and Taraska 2012; Damouni et al. 2025; Goulden et al. 2019; Kalasova et al. 2016; Krishnan et al. 2007; Stilling et al. 2022; Thorn 2017; Toth et al. 2016). Common examples include the PLCδ1-PH domain, used to track PI(4,5)P2 at the plasma membrane, the EEA1-FYVE domain, which binds PI3P on endosomes, or OSH1-PH that recognize PI4P (Gillooly et al. 2000; Lemmon 2008; Shin et al. 2020; Varnai et al. 2002). The molecular biosensors most frequently used in imaging-based methods are summarized in Table 2.

**Table 2:** Phosphoinositide biosensors used in imaging-based methods

Lipid	Molecular biosensor	Reported fusion tags	Imaging-based method	Ref.
PI	*Bc*PI-PLC	GFP SNAP-tag	Confocal	(Maib et al. 2024; Pemberton et al. 2020)
	PI-PLC	mRFP	Confocal	(Kim et al. 2011; Pemberton et al. 2020)
PI(3)P	FYVE(Hrs) × 2	Myc Snap-tag	Confocal STED	(Gaullier et al. 1998; Maib et al. 2024)
	FYVE(EEA1) × 2	GFP Myc	Confocal	(Burd & Emr 1998; Gaullier et al. 1998; Hammond et al. 2014)
	p40^phox^-PX	GFP	Confocal	(Bravo et al. 2001; Ellson et al. 2001; Gozani et al. 2003)
PI(4)P	SidC	Snap-tag	Confocal STED	(Maib et al. 2024)
	SidM-P4M	mCherry GFP iRFP	Confocal	(Hammond et al. 2014)
	SidM-P4M × 2	Cerulean GFP sLuc	BRET Confocal	(Hammond et al. 2014; Pemberton et al. 2020; Sohn et al. 2018; Tóth et al., 2016)
	ORP5-PH	GFP mCherry	Confocal	(Sohn et al. 2018)
	ORP8-PH	GFP mCherry	Confocal TIRF	(Sohn et al. 2016, 2018)
	OSBP-PH	CFP GFP mRFP	Confocal FRET	(Balla et al. 2005)
	FAPP1-PH	CFP GFP mRFP	Confocal FRET	(Balla et al. 2005; Hammond et al. 2014; Levine and Munro 2002)
	OSH2-PH × 2	Cerulean sLuc	BRET Confocal	(Tóth et al., 2016)
PI(5)P	ING2-PHD × 3	GFP Snap-tag	Confocal	(Maib et al. 2024; Pendaries et al. 2006)
	ING2-PHD × 2	GST	Confocal	(Pendaries et al. 2006)
PI(4,5)P2	PLCδ1-PH	Cerulean CFP GFP mCherry sLuc Snap-tag YFP	Confocal FRET BRET TIRF	(Balla et al. 2005; Gozani et al. 2003; Halaszovich et al. 2009; Hammond et al. 2014; Maib et al. 2024; Quinn et al. 2008; Tóth et al., 2016; Várnai and Balla, 1998)
	PLCδ4-PH	GFP	Confocal	(Lee et al. 2004)
	Tubby	GFP YFP	Confocal TIRF	(Halaszovich et al. 2009; Quinn et al. 2008)
PI(3,5)P2	ML1-N × 2	GFP mCherry	Confocal	(Li et al. 2013)
	SnxA	GFP	Confocal	(Vines et al. 2023)
	SnxA-PX × 2	GFP Snap-tag	Confocal	(Maib et al. 2024; Vines et al. 2023)
PI(3,4)P2	TAPP1-cPH × 3	GFP mCherry Snap-tag	Confocal TIRF	(Goulden et al. 2019; Maib et al. 2024)
	TAPP1-PH × 2	GFP sLuc	BRET Confocal	(Damouni et al. 2025; Fernandes et al. 2023)
PI(3,4,5)P3	ARNO-PH	CFP FLAG RFP	Confocal	(Hofmann et al. 2007; Li et al. 2007; Manna et al. 2007)
	ARNO2^G−I303E^-PH × 2	GFP	Confocal TIRF	(Goulden et al. 2019)
	Btk-PH	CFP GFP mVenus sLuc Snap-tag	BRET Confocal	(Damouni et al. 2025; Maib et al. 2024; Manna et al. 2007; Várnai et al., 1999)
	GRP1-PH	CFP GFP sLuc	BRET Confocal	(Damouni et al. 2025; Manna et al. 2007)

BRET, bioluminescence resonance energy transfer; CFP, cyan fluorescent protein; FRET, Förster resonance energy transfer; GFP, green fluorescent protein; GST, glutathione *S*-transferase tag; iRFP, infrared fluorescent protein; mCherry, monomeric Cherry fluorescent protein; mRFP, monomeric red fluorescent protein; sLuc, super-luciferase; TIRF, total internal reflection fluorescence microscopy; YFP, yellow fluorescent protein

Despite their widespread use, recombinant biosensors are associated with several recurring limitations. High expression levels can perturb endogenous phosphoinositide pools through lipid sequestration, aggregation, altered membrane distribution, or nonphysiological interactions, effects that are particularly problematic in quantitative or multiplexed imaging experiments (Chen et al. 2025; Goulden et al. 2019; Kalasova et al. 2016; Maib et al. 2024; Wills et al. 2018). In addition, the size and biochemical properties of the fusion tag can affect biosensor folding and binding behavior, potentially altering affinity or specificity (Crivat and Taraska 2012; Montecinos-Franjola et al. 2020). Fluorescence-based approaches further require careful control of illumination conditions, as fluorescence-based methods that rely on high-intensity illumination are susceptible to photobleaching and phototoxicity (Kiepas et al. 2020). Luminescent biosensors avoid excitation-related artifacts but depend on luciferase substrates, which may introduce cytotoxic effects during extended measurements (Wu and Jiang 2022).

As an alternative to live-cell expression, recombinant biosensors can be purified and applied as exogenous probes to fixed samples, reducing artifacts caused by overexpression while enabling sensitive detection of endogenous phosphoinositide pools (Maib et al. 2024; Palmieri et al. 2010). Antibody-based detection represents another fixed-cell strategy, although validated antibodies are available for only a limited subset of phosphoinositides (Kalasova et al. 2016; Sztacho et al. 2024). However, both of those approaches preclude analysis of lipid dynamics. Overall, biosensors used in live-cell imaging allow analysis of phosphoinositide dynamics but may perturb endogenous lipid organization, whereas biosensors applied in fixed-cell approaches provide stable snapshots of phosphoinositide distribution without temporal resolution. The choice of biosensor should therefore reflect the balance between dynamic resolution and methodological perturbation required by the experimental question.

### Fluorescence-based microscopy methods

**Fig. 3:**
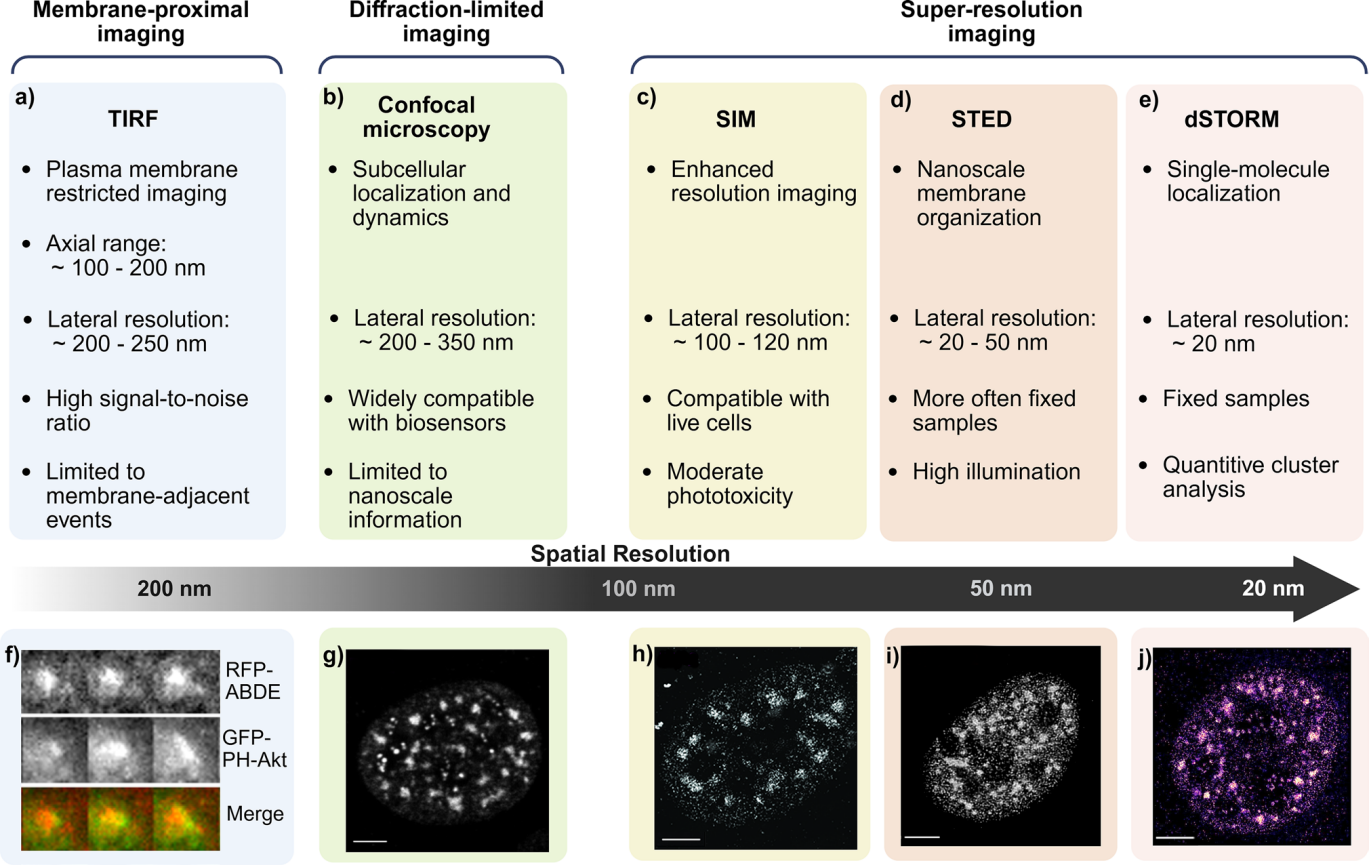
Fluorescence microscopy approaches for phosphoinositide imaging across spatial scales. Fluorescence microscopy techniques for phosphoinositide analysis are arranged by their effective spatial resolution and biological scope. (**a**) TIRF microscopy enables sensitive detection of plasma membrane-restricted lipid dynamics, while (**b**) confocal microscopy provides diffraction-limited imaging across cellular compartments in living cells. Superresolution approaches, such as (**c**) SIM, (**d**) STED, and (**e**) dSTORM microscopy, overcome the diffraction limit to resolve the organization of phosphoinositides at the nanoscale, but this comes at the cost of slower acquisition speeds, increased phototoxicity, and more complex sample preparation. (**f**) Representative TIRF live-cell image of podosomes in cells expressing GFP–PH-Akt to visualize PI(3,4,5)P3 dynamics, with mRFP–ABDE marking actin. Adapted from Sztacho et al. (2016), *PLoS One*. Licensed under CC BY 4.0. (**g**–**j**) Representative images of nuclear PI(4,5)P2 acquired by diffraction-limited and superresolution microscopy in U2OS cells. (**g**) Representative confocal image. Adapted from Balaban et al. 2023, *Biomolecules*. Licensed under CC BY 4.0. (**h**) Representative SIM image. Adapted from Sztacho et al. 2024, *PLoS Genet*. Licensed under CC BY 4.0. (**i**) Representative STED image. Adapted from Balaban et al. 2021, *Cells*. Licensed under CC BY 4.0. (**j**) Representative dSTORM image. Adapted from Hoboth et al. 2021a, with permission from, *MethodsX*. License no. 6240190941369. Scale bars, 5 µm. Open-access works are licensed under the Creative Commons Attribution 4.0 International License (CC BY 4.0; http://creativecommons.org/licenses/by/4.0/). Created in https://BioRender.com

Different fluorescence microscopy approaches provide access to distinct spatial and temporal aspects of phosphoinositide organization within cells (Fig. 3). Confocal microscopy uses a focused laser beam to illuminate a defined point within the sample, and the emitted fluorescence is directed through a pinhole that excludes out-of-focus light, thereby improving image contrast and optical resolution (Atak et al. 2023). This approach provides reliable optical sectioning and supports both two-dimensional imaging and three-dimensional reconstruction, making it well-suited for tracking phosphoinositide dynamics. To resolve finer structural details beyond the resolution limits of confocal microscopy, superresolution techniques extend fluorescence imaging into the nanoscale (Curthoys et al. 2015). Structured illumination microscopy (SIM) achieves resolution of approximately 100—120 nm by using patterned excitation and computational reconstruction to enhance image detail. This technique was used to study the fine-scale clustering of the superenhancer BRD4 in relation to nuclear PI(4,5)P2 levels, showing that changes in PI(4,5)P2 abundance influence BRD4 condensate formation (Sztacho et al. 2024). Another technique, stimulated emission depletion (STED) microscopy, which typically reaches 20—50 nm resolution, improves spatial precision by selectively depleting fluorescence surrounding the focal spot (Curthoys et al. 2015). Combining STED with purified recombinant biosensors enabled visualization of the nanoscale organization of PI3P-positive endosomal tubules and PI4P nanoclusters at the Golgi, while avoiding typical overexpression artifacts (Maib et al. 2024). Single-molecule localization techniques such as direct stochastic optical reconstruction microscopy (dSTORM) provide even higher precision, with ~ 20 nm resolution. dSTORM relies on the controlled blinking of synthetic dyes (e.g., Alexa 647, Cy5) induced by redox-based or light-driven switching. In phosphoinositide research, dual-color quantitative dSTORM (Q-DC-dSTORM) was used to resolve the nuclear distribution of PI(4,5)P2, PI(3,4)P2, and PI4P and showed their enrichment within SON-labeled nuclear speckles (Hoboth et al. 2021a, 2021b). Later, the same approach was used to study the spatial relationships between PI(4,5)P2 and PI(3,4)P2, and the dynamic regulation of RNA transcription (Hoboth et al. 2024).

Although high illumination intensities can cause photobleaching and phototoxicity, superresolution microscopy enables analysis of phosphoinositide organization at spatial scales not accessible by confocal imaging. Confocal and SIM are readily combined with genetically encoded phosphoinositide biosensors in live cells, whereas STED and single-molecule localization approaches often rely on purified probes or fixed samples to minimize overexpression artifacts. Beyond imaging strategies that primarily resolve spatial organization, complementary microscopy-based techniques emphasize phosphoinositide dynamics, membrane-proximal behavior, and protein–lipid interactions, including total internal reflection fluorescence (TIRF) microscopy, fluorescence recovery after photobleaching (FRAP), and biosensor-based Förster and bioluminescence resonance energy transfer (FRET and BRET) approaches.

#### Total internal reflection fluorescence (TIRF)

TIRF microscopy offers a selective approach to visualize phosphoinositide dynamics near or at the plasma membrane. By generating a shallow evanescent field at the interface, TIRF selectively excites fluorophores within approximately 100 nm—200 nm of the membrane, substantially reducing background fluorescence. This selective illumination enhances the signal-to-noise ratio and limits phototoxicity, making TIRF well-suited for monitoring membrane-proximal phosphoinositide-dependent events and interactions involving lipid-binding biosensors such as PH domains. In addition, the use of sensitive charge-coupled device (CCD) detectors enables quantitative comparison of membrane-associated fluorescence signals through pixel intensity analysis (Fish 2022; Mattheyses et al. 2010; Varnai et al. 2017).

TIRF has proven particularly useful in studies exploring the spatial organization of phosphoinositides at the plasma membrane. One illustrative example is oxysterol-binding protein-related protein 8 (ORP8), an endoplasmic reticulum—plasma membrane tether whose recruitment relies on PI4P binding, was visualized by TIRF as discrete puncta corresponding to ER–PM contact sites. Under conditions of altered lipid composition or acute inhibition of PI4KA using the small-molecule inhibitor A2, these puncta were markedly reduced or lost, consistent with reduced PI4P availability at the plasma membrane. This work illustrates how TIRF-based imaging of phosphoinositide-binding proteins can be used to visualize the effects of small-molecule perturbations on local phosphoinositide pools at membrane contact sites (Sohn et al. 2016). Despite these strengths, TIRF microscopy is inherently restricted to the plasma membrane and provides limited information on phosphoinositide pools in intracellular compartments such as endosomes, the Golgi, or the nucleus, necessitating complementary imaging approaches for a comprehensive analysis of lipid distribution.

#### Förster resonance energy transfer (FRET)

**Fig. 4:**
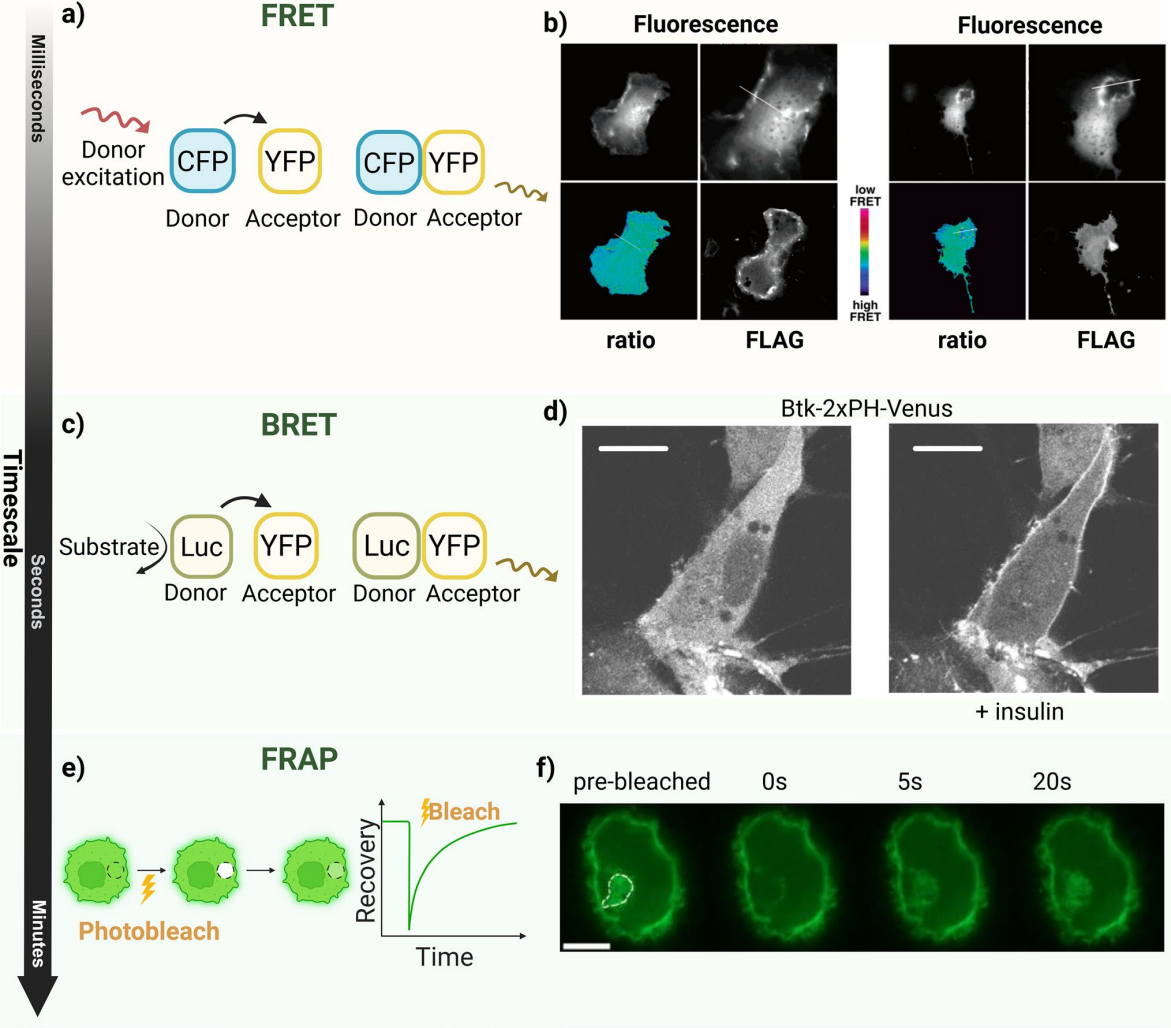
Principles of FRET, BRET, and FRAP used to assess molecular interactions and dynamics. (**a**) Schematic principle of fluorescence resonance energy transfer (FRET). Energy transfer occurs from an excited donor fluorophore to a nearby acceptor fluorophore, enabling detection of molecular proximity and interactions. (**b**) FRET ratio imaging of the CAY phosphoinositide sensor in Swiss 3T3 fibroblasts. Expression of the Akt PH domain suppresses FRET loss (left), whereas the PLC-δ PH domain does not suppress FRET loss (right), indicating lipid-dependent signaling in membrane ruffles. Adapted from Cicchetti et al. 2004, with permission from *Biochemistry*. License no. 6230751337527. (**c**) Schematic principle of bioluminescence resonance energy transfer (BRET), where energy generated by luciferase oxidation of its substrate is transferred to a fluorescent acceptor. (**d**) Representative confocal images from a BRET-based phosphoinositide analysis of HEK293A cells expressing the Btk-2xPH-Venus PI(3,4,5)P3 biosensor. Insulin stimulation promotes plasma membrane recruitment of the biosensor, reflecting increased PI(3,4,5)P3 levels. Adapted from Damouni et al. 2025, with permission from *J Biol Chem*. License no. 6231240566892. (**e**) Schematic overview of fluorescence recovery after photobleaching (FRAP). A defined region is photobleached, and fluorescence recovery is monitored over time. (**f**) Representative FRAP analysis of the PI(4,5)P2 probe PH-GFP in intracellular plasma membrane-connected compartments (IPMCs) of monocyte-derived macrophages (MDMs). A defined IPMC region was photobleached, and representative images were acquired before bleaching and at 0, 5, and 20 s after bleaching. Adapted from Mlcochova et al. 2013, *BMC Biol*. Licensed under CC BY 2.0. Scale bars, 10 μm. Open-access work is licensed under the Creative Commons Attribution 2.0 International License (CC BY 2.0; https://creativecommons.org/licenses/by/2.0/). Created in  https://BioRender.com

While TIRF focuses on events occurring at the plasma membrane, FRET enables the real-time mapping of molecular interactions between proteins, lipids, and biosensor domains throughout the cell (Fig. 4a) (Algar et al. 2019; Broussard and Green 2017). FRET is based on nonradiative energy transfer between a donor and acceptor fluorophore with overlapping spectra and is strongly distance-dependent, making it well-suited for reporting conformational changes or binding events within biosensor constructs. The efficiency of this transfer, and the resulting acceptor fluorescence, increases as the distance between the donor and acceptor becomes smaller. In phosphoinositide research, FRET measurements typically rely on overexpressed recombinant fluorescent donor–acceptor biosensor pairs. However, the choice and localization of fluorescent tags must be carefully considered to minimize perturbation of protein structure, function, or lipid organization.

An early example of this approach is the CAY biosensor, which uses a CFP-YFP fluorescent protein donor–acceptor pair linked by a phosphoinositide-binding peptide derived from the ActA protein of *Listeria monocytogenes*. Phosphoinositide binding induced a conformational change that increased fluorophore separation and reduced FRET efficiency. While CAY enabled visualization of localized phosphoinositide enrichment in dynamic membrane regions such as ruffles, its broad reactivity toward multiple phosphorylated phosphoinositides limited lipid specificity and sensitivity to small or compartmentalized pools, including nuclear phosphoinositides (Cicchetti et al. 2004). Similarly, the commonly used CFP/YFP FRET pair was applied in a system designed to monitor PI(4,5)P2 dynamics at the plasma membrane. This system combined ECFP- and EYFP-tagged PH-PLCδ1 domains with the voltage-sensitive phosphatase Ci-VSP in a tricistronic construct separated by 2A-peptides (Hertel et al. 2011). During translation, those peptides support a self-cleavage that leads to the production of the three proteins in equimolar amounts and limits the number of necessary transfections (Szymczak et al. 2004). By co-expressing the biosensor components with the voltage-sensitive phosphatase Ci-VSP, this design enabled controlled enzymatic depletion and recovery of PI(4,5)P2 without small-molecule inhibitors (Hertel et al. 2011). More recently, genetically encoded FRET-based biosensors with enhanced specificity for PI(3,4)P2 and PI(3,4,5)P3 were developed and targeted to defined subcellular compartments. These probes were enabled monitoring of stimulus-dependent phosphoinositide redistribution between the plasma membrane and intracellular organelles. In particular, PI(3,4,5)P3 exhibited a more persistent lysosomal signal compared with the transient behavior of PI(3,4)P2, supporting the existence of lipid-specific intracellular pools (Sahan et al. 2025).

Taken together, FRET-based phosphoinositide biosensors have evolved from broadly reactive reporters of lipid enrichment to more selective, compartment-targeted tools capable of resolving differences in phosphoinositide behavior across cellular locations. While their sensitivity to nanoscale molecular proximity makes them powerful for probing phosphoinositide-associated signaling, FRET measurements remain subject to limitations such as spectral overlap, photobleaching, and perturbation of endogenous lipid pools due to biosensor expression, which must be carefully considered in experimental design.

#### Bioluminescence resonance energy transfer (BRET)

In contrast to FRET, which requires external excitation of the donor fluorophore, BRET uses a bioluminescent donor, thereby avoiding the background fluorescence, photobleaching, and phototoxicity associated with illumination (Fig. 4b) (Xu et al. 1999). In BRET, bioluminescence is generated by a luciferase enzyme that emits light upon reacting with its substrate, and this emission can excite a nearby acceptor fluorophore if their spectra overlap. This excitation-independent mechanism results in low background and reduced autofluorescence, making BRET particularly well-suited for monitoring phosphoinositide-dependent signaling under conditions where minimal light exposure is required (El Khamlichi et al. 2019; Pfleger and Eidne 2006).

To address the variability and limited quantifiability of small phosphoinositide changes in fluorescence-based imaging, a BRET-based biosensor strategy was developed to enable robust population-level measurements. In this system, a luciferase-tagged phosphoinositide-binding domain acts as the donor, while a membrane-anchored Venus fluorophore serves as the acceptor (Damouni et al. 2025; Toth et al. 2016). Reliable intermolecular BRET depends on balanced donor–acceptor expression. To achieve this, both components were encoded within a single plasmid separated by a T2A peptide. Using this BRET-based biosensor design, the authors demonstrated sensitive detection of hormone-induced phosphoinositide changes at the plasma membrane, revealing PKC-dependent activation of PI4KA during PLC signaling (Toth et al. 2016).

Although BRET avoids many artifacts associated with illumination, it also introduces specific limitations, including the need for continuous substrate supply, variability in substrate availability, and potential cytotoxicity during extended measurements. Nevertheless, BRET provides a valuable complement to FRET-based approaches, particularly for applications requiring low background and reduced phototoxicity.

#### Fluorescence recovery after photobleaching (FRAP)

In addition to approaches that report molecular interactions and lipid recruitment, phosphoinositide-dependent processes can also be examined by analyzing molecular mobility and exchange within cellular structures. FRAP is a microscopy-based approach used to study how molecules move and interact in living cells. In FRAP experiments, fluorescently tagged proteins, often recombinant biosensors in phosphoinositide research, are irreversibly photobleached within a defined region using a focused laser beam, and fluorescence recovery results from diffusion of unbleached molecules into the bleached area. The kinetics of this recovery reflect molecular mobility as well as transient binding interactions that slow diffusion until dissociation occurs (Fig. 4c) (Lippincott-Schwartz et al. 2018).

A representative application of FRAP in phosphoinositide research examined how phosphoinositide binding influences the dynamics of the actin-binding protein α-actinin. Cells expressing GFP-tagged wild-type α-actinin or a mutant with reduced phosphoinositide affinity were photobleached at stress fibers and focal adhesions. The mutant displayed markedly slower fluorescence recovery than the wild type, indicating that phosphoinositide binding modulates the association–dissociation kinetics of α-actinin with actin filaments rather than simply its localization (Fraley et al. 2005).

FRAP has also been applied to investigate the membrane-binding behavior of phosphoinositide-recognition domains themselves. In neuroblastoma cells expressing two fluorescently tagged PI(4,5)P2 biosensors, GFP-PLCδ4-PH and GFP-PLCδ1-PH, fluorescence recovery profiles revealed distinct exchange kinetics at the plasma membrane. The PLCδ4-PH domain exhibited faster recovery despite lower membrane affinity than PLCδ1-PH, consistent with a more transient interaction with PI(4,5)P2. This study illustrates how FRAP can resolve subtle differences in the dynamic interplay between phosphoinositides and their binding domains that are not evident from static imaging alone (Lee et al. 2004).

In summary, FRAP provides a quantitative framework for analyzing phosphoinositide-dependent protein turnover and exchange within cellular structures. The method is well-suited for estimating mobility and distinguishing between bound and diffusible populations, but its spatial resolution is limited, recovery curves may reflect multiple kinetic processes, and it does not directly measure lipid dynamics. Consequently, careful experimental design and complementary approaches are required for accurate interpretation.

## Conclusions

Phosphoinositides are essential regulators of signal transduction, membrane dynamics, gene expression, and cell survival, and their dysregulation is implicated in a wide range of diseases, including cancer (Balaban et al. 2023; Balla 2013; Castano et al. 2019; Raghu et al. 2019; Saarikangas et al. 2010; Vidalle et al. 2023). Their low abundance, rapid turnover, and highly compartmentalized distribution make them intrinsically challenging to study and require experimental strategies that combine high spatial and temporal resolution with minimal perturbation of endogenous lipid pools. A broad range of complementary approaches are available to manipulate, visualize, and quantify phosphoinositides. Acute perturbation using small-molecule inhibitors has been instrumental in dissecting pathway dependencies and turnover kinetics, although limited selectivity and compensatory responses can complicate mechanistic interpretation (Costa et al. 2015; Gharbi et al. 2007; Lannutti et al. 2011; Workman et al. 2010). Genetic and inducible strategies, including RNAi, CRISPR–Cas9-mediated editing, and chemically or optogenetically controlled enzymes, enable isoform-specific and spatially restricted manipulation and reveal longer-term adaptive responses that are not accessible through acute inhibition alone (Barrangou et al. 2015; Caires et al. 2021; Lampreht Tratar et al. 2018; Moore et al. 2010; Subramanya et al. 2010; Sun et al. 2011). Complementary visualization and quantification strategies provide further insight into phosphoinositide localization, dynamics, and molecular diversity. Fluorescent biosensors combined with advanced imaging link lipid dynamics to cellular behaviors, while confocal and superresolution microscopy resolve spatial organization down to the nanoscale (Crivat and Taraska 2012; Curthoys et al. 2015; Hoboth et al. 2021a, 2021b). In parallel, biochemical techniques such as TLC and MS provide quantitative measurements of phosphoinositide abundance, acyl-chain composition, and positional isomers (DeLong et al. 2001; Engel and Schiller 2021; Li & Lammerhofer 2021; Serunian et al. 1991; Wenk et al. 2003).

Despite their strengths, all approaches have inherent limitations. Recombinant biosensors can sequester their targets, inhibitors often affect multiple pathways, genetic perturbations may induce nonphysiological lipid levels, and even advanced lipidomics can miss rare species or spatial information (Gharbi et al. 2007; Kalasova et al. 2016; Kielkowska et al. 2014; Knight et al. 2006; Li and Lammerhofer 2021; Maib et al. 2024; Wills et al. 2018; Workman et al. 2010). Consequently, no single method captures phosphoinositide signaling in its entirety.

Integrated and multimodal strategies are therefore essential to overcome these limitations. Rapid and transient lipid signaling is best addressed by combining acute inhibition with high-resolution imaging, whereas isoform-specific functions and long-term adaptations benefit from genetic approaches. Questions related to lipid diversity and metabolism rely on quantitative biochemical approaches. Robust conclusions are obtained when observations are supported by multiple independent methodologies, such as integrating chemical and genetic manipulation with imaging and lipidomics. By emphasizing tools that directly link manipulation of phosphoinositide levels to functional outcomes, this review highlights phosphoinositides as integrated regulators embedded within complex cellular networks. A multimodal experimental framework is therefore crucial for defining how distinct phosphoinositide species function within specific compartments and how their localized dynamics shape cellular physiology and disease.

## Data Availability

No datasets were generated or analyzed during the current study.

## Declarations

### Informed content

During the preparation of this work the authors used DeepL and ChatGTP to improve the readability and language of the manuscript. After using this tool, the authors reviewed and edited the content as needed and take full responsibility for the content of the published article.
